# Supplementary material for: Beyond the French Flag Model: Exploiting Spatial and Gene Regulatory Interactions for Positional Information
Source: PLoS One. 2016 Sep 27;11(9):e0163628. doi: 10.1371/journal.pone.0163628 (PMC5038966; doi:10.1371/journal.pone.0163628)
Supplement: S1 Appendix — A lower bound on the error of a positional estimator with limited positional information is derived. (PDF) [file pone.0163628.s001.pdf]

Bounding position estimation error by positional information

Patrick Hillenbrand, Ulrich Gerland, and Gašper Tkačik

We consider a one-dimensional lattice with N lattice sites, where each site can be thought of as a row or a ring of nuclei in an embryo. We define positional information as $I(x;\vec{g})$, where I denotes mutual information, $x \in \{1,...,N\}$ the discrete position and $\vec{g}$ is a vector of morphogen concentrations at a specific lattice site. A cell or nucleus can estimate its position x in the embryo by reading out the morphogen concentrations $\vec{g}$. Such an estimator $\hat{x}$ is part of a Markov chain $x \to \vec{g} \to \hat{x}$. The variance of $\hat{x}$ is defined as $\mathrm{var}(\hat{x}) = E(x-\hat{x})^2$, where E denotes the expectation value. Assuming that we can calculate $I(x;\vec{g})$, we want to find a lower bound for the variance of the estimator in the form $var(\hat{x}) \geq f[I(x;\vec{g})]$, where f is a function yet to be determined. The following derivation closely follows the proof for Fano's inequality for continuous variables in [1].

We assume that a cell or nucleus has no prior information about its position, i.e. without knowledge of $\vec{g}$, x follows a uniform distribution $x \sim 1/N$. We note that this distribution has the variance $\mathrm{var}(x) = (N^2-1)/12$ and entropy in bits $H(x) = \log_2(N)$. Therefore, after some simple transformations, the variance can be written as $\mathrm{var}(x) = (2^{2H(x)}-1)/12$. The best estimator for a random variable is its expectation value and thus

$$\mathrm{var}(\hat{x}) = E(x-\hat{x})^2 \geq E(x-E(x))^2 = \mathrm{var}(x) = \frac{1}{12}\left(2^{2H(x)}-1\right) . \tag{1}$$

Additional information can only decrease the entropy ($H(x|\vec{g}) \leq H(x)$), which leads to

$$\mathrm{var}(\hat{x}) \geq \frac{1}{12}\left(2^{2H(x|\vec{g})}-1\right) . \tag{2}$$

Finally, we use the definition of mutual information $I(x;\vec{g}) = H(x) - H(x|\vec{g})$ to write $H(x|\vec{g}) = H(x) - I(x;\vec{g}) = log_2(N) - I(x;\vec{g})$, where we used the entropy of the uniform prior distribution of x. This leads to the final inequality

$$\mathrm{var}(\hat{x}) \geq \frac{1}{12}\left(N^2 2^{-2I(x;\vec{g})}-1\right) . \tag{3}$$

Essentially the same arguments can be applied if the x is a continuous variable in $[0,L]$, which leads to the inequality

$$\mathrm{var}(\hat{x}) \geq \frac{L^2}{6} 2^{-I(x;\vec{g})} . \tag{4}$$

If x is discrete and $I(x;\vec{g})$ is high enough to distinguish between each lattice site, i.e. $I(x;\vec{g}) \geq \log_2(N)$, than $(N^2 2^{-2I(x;\vec{g})}-1) \leq 0$ and therefore an estimator is not limited by insufficient information – as expected.

[1] Thomas M. Cover and Joy A. Thomas. *Elements of Information Theory (Wiley Series in Telecommunications and Signal Processing)*. Wiley-Interscience, 2006.
