# Supplementary material for: Beyond the French Flag Model: Exploiting Spatial and Gene Regulatory Interactions for Positional Information
Source: PLoS One. 2016 Sep 27;11(9):e0163628. doi: 10.1371/journal.pone.0163628 (PMC5038966; doi:10.1371/journal.pone.0163628)
Supplement: S2 Appendix — The effect of noise in the input field on an Ising model is approximated. Furthermore, methods to compute positional information in an Ising model by transfer matrices and Monte Carlo sampling are outlined. (PDF) [file pone.0163628.s002.pdf]

Computation of positional information in an Ising model

Patrick Hillenbrand, Ulrich Gerland, and Gašper Tkačik

We describe here how positional information $I(\sigma, x)$ can be computed in an Ising model with a inhomogeneous, fluctuating input field. The effects of the input field fluctuations are approximated by expanding the position dependent probability $P(\sigma|x)$ to second order in the input field. In the first section we discuss this expansion for a system with a single spin per lattice site, where the probability $P(\sigma|x)$ can be uniquely described by the position dependent mean spin $\langle\sigma_x\rangle$. The second section generalizes this calculation to K spins per lattice site. The third section describes how $I(\sigma, x)$ can be efficiently computed with the use of transfer matrices. In the last section, the Monte-Carlo simulation for systems with long range interactions and our stochastic optimization are explained.

One spin per lattice site

In our model we assume a lattice with N sites indexed by the position variable x. At each lattice site, there is an input field h_x, which depends on the morphogen field m_x (see main text): $h_x = f(m_x)$. The field h_x has mean values μ_x and fluctuations $\mathrm{var}(h_x) = v_x^2$. h_x acts locally on the spin $\sigma_x \in \{+1, -1\}$, or for short $\sigma_x \in \{+, -\}$. Following the standard Ising model, the Hamiltonian can then be written as

$$H(\vec{\sigma}) = \sum_{x=1}^{N} -J\sigma_x\sigma_{x+1} - h_x\sigma_x \,, \tag{1}$$

where the parameter J denotes the spatial spin-spin coupling (for simplicity we define an additional spin $\sigma_{N+1} = 0$). The probability of a certain state of the spin lattice $\vec{\sigma}$, given a fixed input field $\vec{h}$ is

$$P(\vec{\sigma}|\vec{h}) = \frac{e^{-\beta H(\vec{\sigma})}}{Z} = \frac{e^{-\beta H(\vec{\sigma})}}{\sum_{\vec{\sigma}} e^{-\beta H(\vec{\sigma})}} \,, \tag{2}$$

where β is the inverse of the intrinsic noise level η introduced in the main text.

We define positional information as

$$I(\sigma; x) = \sum_{x=1}^{N} P(x) \sum_{\sigma=\pm 1} P(\sigma|x) \log\left[\frac{P(\sigma|x)}{P(\sigma)}\right] = \frac{1}{N}\sum_{x=1}^{N}\sum_{\sigma=\pm 1} P(\sigma|x) \log\left[\frac{P(\sigma|x)}{P(\sigma)}\right] \,. \tag{3}$$

We assume a uniform prior distribution for the location $P(x) = 1/N$. Then the marginal distribution for a spin state is

$$P(\sigma) = (1/N)\sum_{x=1}^{N} P(\sigma|x) \,. \tag{4}$$

The conditional probability $P(\sigma|x)$ can be calculated by marginalizing over the input field $\vec{h}$ and over all possible states of the spins except σ_x (we denote the corresponding summation by $\sum_{\vec{\sigma}/\sigma_x}$):

$$P(\sigma|x) = \sum_{\vec{\sigma}/\sigma_x} P(\vec{\sigma}|\vec{x}) = \sum_{\vec{\sigma}/\sigma_x} \int d\vec{h}\, P(\vec{h}|\vec{x}) P(\vec{\sigma}|\vec{h}) \,. \tag{5}$$

In the case of one spin per lattice site, we can write $\langle\sigma_x\rangle = P(+|x) - P(-|x) = P(+|x) - (1 - P(+|x)) = 2P(+|x) - 1$ and thus $P(+|x) = (1/2)(\langle\sigma_x\rangle + 1)$. Therefore, if we can calculate $\langle\sigma_x\rangle$, we can calculate $I(\sigma; x)$ by

$$I(\sigma; x) = \frac{1}{N}\sum_{i=1}^{N}\left[P(+|x)\log\left[\frac{P(+|x)}{P(+)}\right] + (1 - P(+|x))\log\left[\frac{1 - P(+|x)}{1 - P(+)}\right]\right] \,. \tag{6}$$

We can write $\langle\sigma_x\rangle$ as

$$\langle\sigma_x\rangle = \sum_{\vec{\sigma}} \sigma_x P(\vec{\sigma}|\vec{x}) = \sum_{\vec{\sigma}} \sigma_x \int d\vec{h} P(\vec{h}) P(\vec{\sigma}|\vec{h}) \,. \tag{7}$$

If we assume $P(\vec{h})$ to be independent at each lattice site ($P(\vec{h}) = \prod_j P(h_j)$) and sharply peaked around its mean value $\langle\vec{h}\rangle = \vec{\mu}$ with variance $\mathrm{var}(h_y) = v_y^2$, we can expand $P(\vec{\sigma}|\vec{h})$ to second order around $\vec{\mu}$:

$$\langle\sigma_x\rangle \approx \sum_{\vec{\sigma}} \sigma_x \int d\vec{h}\, P(\vec{h}) \left[P(\vec{\sigma}|\vec{\mu}) + \sum_y \left.\frac{\partial P(\vec{\sigma}|\vec{h})}{\partial h_y}\right|_{\vec{\mu}} (h_y - \mu_y) + \frac{1}{2}\sum_{yz} \left.\frac{\partial^2 P(\vec{\sigma}|\vec{h})}{\partial h_y \partial h_z}\right|_{\vec{\mu}} (h_y - \mu_y)(h_z - \mu_z) \right] . \quad (8)$$

With $\int d\vec{h} P(\vec{h})(h_j - \mu_j) = (\mu_j - \mu_j) = 0$ and $\int d\vec{h} P(\vec{h})(h_y - \mu_y)(h_z - \mu_z) = \mathrm{cov}(h_y, h_z) = \delta_{yz} v_y^2$ this yields

$$\langle\sigma_x\rangle \approx \sum_{\vec{\sigma}} \sigma_x \left[P(\vec{\sigma}|\vec{\mu}) + \frac{1}{2}\sum_y v_y^2 \left.\frac{\partial^2 P(\vec{\sigma}|\vec{h})}{\partial h_y^2}\right|_{\vec{\mu}} \right] , \quad (9)$$

$$= \langle\sigma_x\rangle_{\vec{\mu}} + \frac{1}{2}\sum_y v_y^2 \left[\sum_{\vec{\sigma}} \sigma_x \left.\frac{\partial^2 P(\vec{\sigma}|\vec{h})}{\partial h_y^2}\right|_{\vec{\mu}} \right] , \quad (10)$$

where $\langle\sigma_x\rangle_{\vec{\mu}} = \sum_{\vec{\sigma}} \sigma_x P(\vec{\sigma}|\vec{\mu})$ is the mean spin at position x in response to the mean input field $\vec{\mu}$. We now must compute the derivative:

$$\frac{\partial^2 P(\vec{\sigma}|\vec{h})}{\partial h_y^2} = \frac{\partial}{\partial h_y} \left(-\frac{1}{Z^2} e^{-\beta H} \frac{\partial Z}{\partial h_y} - \beta \frac{\partial H}{\partial h_y} \frac{e^{-\beta H}}{Z} \right) \quad (11)$$

$$= \frac{\partial}{\partial h_y} \left(-\frac{1}{Z^2} e^{-\beta H} \left(\sum_{\vec{\sigma}} \beta\sigma_y e^{-\beta H} \right) + \beta\sigma_y \frac{e^{-\beta H}}{Z} \right) \quad (12)$$

$$= \frac{2}{Z^3}\frac{\partial Z}{\partial h_y} e^{-\beta H} \left(\sum_{\vec{\sigma}} \beta\sigma_y e^{-\beta H} \right) - \frac{e^{-\beta H}}{Z^2} \beta\sigma_y \left(\sum_{\vec{\sigma}} \beta\sigma_y e^{-\beta H} \right) - \frac{e^{-\beta H}}{Z^2} \left(\sum_{\vec{\sigma}} (\beta\sigma_y)^2 e^{-\beta H} \right) \quad (13)$$

$$+\beta\sigma_y \left(-\frac{e^{-\beta H}}{Z^2} \sum_{\vec{\sigma}} \beta\sigma_y e^{-\beta H} + \beta\sigma_y \frac{e^{-\beta H}}{Z} \right) \quad (14)$$

$$= 2\beta^2 P(\vec{\sigma}|\vec{h}) \langle\sigma_y\rangle^2 - \beta^2 P(\vec{\sigma}|\vec{h}) \sigma_y \langle\sigma_y\rangle - \beta^2 P(\vec{\sigma}|\vec{h}) - \beta^2 P(\vec{\sigma}|\vec{h}) \sigma_y \langle\sigma_y\rangle + \beta^2 P(\vec{\sigma}|\vec{h}) \quad (15)$$

$$= 2\beta^2 P(\vec{\sigma}|\vec{h}) \left[\langle\sigma_y\rangle^2 - \sigma_y \langle\sigma_y\rangle \right] \quad (16)$$

Putting this back into eq. 10 yields

$$\langle\sigma_x\rangle \approx \langle\sigma_x\rangle_{\vec{\mu}} + \beta^2 \sum_y v_y^2 \left[\sum_{\vec{\sigma}} \sigma_x P(\vec{\sigma}|\vec{\mu}) (\langle\sigma_j\rangle_{\vec{\mu}}^2 - \sigma_y \langle\sigma_y\rangle_{\vec{\mu}}) \right] \quad (17)$$

$$= \langle\sigma_x\rangle_{\vec{\mu}} - \beta^2 \sum_y v_y^2 \langle\sigma_y\rangle_{\vec{\mu}}\ \mathrm{cov}(\sigma_x, \sigma_y)_{\vec{\mu}} \quad (18)$$

where an index $\vec{\mu}$ denotes that expectation values are taken at mean input field.

The approximation is valid if $P(\vec{\sigma}|\vec{h})$ does not exhibit much variation across the peak of $P(\vec{h})$. The higher the intrinsic noise level $\eta = 1/\beta$, the less effect do fluctuations in the input field have on the spins. Thus, β can be seen as the sensitivity of the spin lattice to input fluctuations. Therefore, if the product of sensitivity and input fluctuations (βv) is small, the correction term in eq. 18 remains small and in the approximation is valid.

K spins per lattice site

In the case of multiple (K) spins per lattice site we assume that spins of different rows interact locally (coupling strength denoted by $J_{\alpha\beta}$), whereas spins of the same row interact spatially with their nearest neighbors (coupling strength denoted by J_α, J_β, ...). We denote the state of a single spin at lattice site x and row α as σ_x^α. A specific state of all spins at a lattice site x is denoted as $\boldsymbol{\sigma}_x$ and the state of the entire spin lattice as $\vec{\boldsymbol{\sigma}}$. To take into account that the morphogen field m_x acts differently on each row of spins, we define the effective fields $h_x^\alpha = f_\alpha(h_x)$, which

act only their respective spin row: $H = I_{coupling} - \sum_{i=1}^{N}\sum_{\alpha=1}^{K} h_x^\alpha \sigma_x^\alpha$. $I_{coupling}$ sums up all spin-spin couplings in the system:

$$I_{coupling} = -\sum_{x=1}^{N}\left[\sum_{\alpha=1}^{K}\sum_{\beta=\alpha+1}^{K} J_{\alpha\beta}\sigma_x^\alpha\sigma_x^\beta + \sum_{\alpha=1}^{K} J_\alpha \sigma_x^\alpha \sigma_{x+1}^\alpha\right] . \tag{19}$$

Since the effective fields h_x^α at a certain lattice site are all functions of the same (fluctuating) field, their fluctuations are perfectly correlated. Thus, the covariances at a lattice site x are $\mathrm{cov}(h_x^\alpha, h_x^\beta) = v_x^\alpha v_x^\beta$, where v_x^α denotes the standard deviation of the field h_x^α.

Analogous to the 1D case, we can calculate the positional information as

$$I(\vec{\boldsymbol{\sigma}}; x) = \frac{1}{N}\sum_{i=1}^{N}\sum_{\vec{\boldsymbol{\sigma}}} P(\boldsymbol{\sigma}|x)\log\left[\frac{P(\boldsymbol{\sigma}|x)}{P(\boldsymbol{\sigma})}\right] . \tag{20}$$

The second sum runs over all 2^K possible states the spins at a single lattice site can assume. Thus, we must calculate $P(\boldsymbol{\sigma_x}|x)$ for all 2^K different states $\boldsymbol{\sigma_x}$. In comparison to the 1D case, we cannot calculate the conditional probabilities $P(\boldsymbol{\sigma}|x)$ from the mean spins anymore, so we have to execute the marginalization over all lattice sites except for x explicitly. Analogous to the approximation above, we calculate

$$P(\boldsymbol{\sigma}|x) = \sum_{\vec{\boldsymbol{\sigma}}/\boldsymbol{\sigma}_x}\int \boldsymbol{d\vec{h}}\, P(\vec{\boldsymbol{h}})P(\vec{\boldsymbol{\sigma}}|\vec{\boldsymbol{h}}) \approx \sum_{\vec{\boldsymbol{\sigma}}/\boldsymbol{\sigma}_x}\left[P(\vec{\boldsymbol{\sigma}}|\vec{\boldsymbol{h}}) + \frac{1}{2}\sum_{y,(\alpha,\beta)}\mathrm{cov}(h_y^\alpha, h_y^\beta)\left.\frac{\partial^2 P(\vec{\boldsymbol{\sigma}}|\vec{\boldsymbol{h}})}{\partial h_y^\alpha\,\partial h_y^\beta}\right|_{\vec{\boldsymbol{\mu}}}\right] , \tag{21}$$

where we have taken into account that fields across lattice sites are uncorrelated. Again, we must compute the derivative:

$$\frac{\partial^2 P(\vec{\boldsymbol{\sigma}}|\vec{\boldsymbol{h}})}{\partial h_y^\alpha\,\partial h_y^\beta} = \frac{\partial}{\partial h_y^\beta}\left(-\frac{1}{Z^2}\left(\frac{\partial Z}{\partial h_y^\alpha}\right)e^{-\beta H} + \frac{e^{-\beta H}}{Z}(\beta\sigma_y^\alpha)\right) \tag{22}$$

$$= \frac{\partial}{\partial h_y^\beta}\left(-\frac{e^{-\beta H}}{Z^2}\left(\sum_{\vec{\boldsymbol{\sigma}}}\beta\sigma_y^\alpha e^{-\beta H}\right) + \frac{e^{-\beta H}}{Z}(\beta\sigma_y^\alpha)\right) \tag{23}$$

$$= \beta\sigma_y^\alpha\left(\beta\sigma_y^\beta P(\vec{\boldsymbol{\sigma}}|\vec{\boldsymbol{h}}) - \beta P(\vec{\boldsymbol{\sigma}}|\vec{\boldsymbol{h}})\langle\sigma_y^\beta\rangle\right) + 2\frac{e^{-\beta H}}{Z^3}\left(\frac{\partial Z}{\partial h_y^\beta}\right)\left(\sum_{\vec{\boldsymbol{\sigma}}}\beta\sigma_y^\alpha e^{-\beta H}\right) \tag{24}$$

$$-\frac{e^{-\beta H}}{Z}(\beta\sigma_y^\beta)(\beta\langle\sigma_y^\alpha\rangle) - \frac{e^{-\beta H}}{Z^2}\left(\sum_{\vec{\boldsymbol{\sigma}}}\beta^2\sigma_y^\alpha\sigma_y^\beta e^{-\beta H}\right) \tag{25}$$

$$= \beta^2 P(\vec{\boldsymbol{\sigma}}|\vec{\boldsymbol{h}})\left[\sigma_y^\alpha\sigma_y^\beta - \sigma_y^\alpha\langle\sigma_y^\beta\rangle - \sigma_y^\beta\langle\sigma_y^\alpha\rangle + 2\langle\sigma_y^\alpha\rangle\langle\sigma_y^\beta\rangle - \langle\sigma_y^\alpha\sigma_y^\beta\rangle\right] \tag{26}$$

Putting this result back into eq. 21 leads to

$$P(\boldsymbol{\sigma}|x) \tag{27}$$

$$\approx \sum_{\vec{\boldsymbol{\sigma}}/\boldsymbol{\sigma}_x} P(\vec{\boldsymbol{\sigma}}|\vec{\boldsymbol{\mu}}) + \frac{\beta^2}{2}\sum_{y,(\alpha,\beta)}\mathrm{cov}(h_y^\alpha, h_y^\beta)\sum_{\vec{\boldsymbol{\sigma}}/\boldsymbol{\sigma}_x} P(\vec{\boldsymbol{\sigma}}|\vec{\boldsymbol{\mu}})\left[\sigma_y^\alpha\sigma_y^\beta - \sigma_y^\alpha\langle\sigma_y^\beta\rangle - \sigma_y^\beta\langle\sigma_y^\alpha\rangle + 2\langle\sigma_y^\alpha\rangle\langle\sigma_y^\beta\rangle - \langle\sigma_y^\alpha\sigma_y^\beta\rangle\right] \tag{28}$$

$$= P(\boldsymbol{\sigma}_x|\vec{\boldsymbol{\mu}}) + \frac{\beta^2}{2}\sum_{y,(\alpha,\beta)}\mathrm{cov}(h_y^\alpha, h_y^\beta)\left[\langle\sigma_y^\alpha\sigma_y^\beta\rangle_{\boldsymbol{\sigma}_x} - \langle\sigma_y^\alpha\rangle_{\boldsymbol{\sigma}_x}\langle\sigma_y^\beta\rangle - \langle\sigma_y^\beta\rangle_{\boldsymbol{\sigma}_x}\langle\sigma_y^\alpha\rangle + P(\boldsymbol{\sigma}_x|\vec{\boldsymbol{\mu}})\left(2\langle\sigma_y^\alpha\rangle\langle\sigma_y^\beta\rangle - \langle\sigma_y^\alpha\sigma_y^\beta\rangle\right)\right] \tag{29}$$

Here, $P(\boldsymbol{\sigma}_x|\vec{\boldsymbol{\mu}})$ is the conditional probability at mean input field. Mean values with an index $\langle\cdot\rangle_{\boldsymbol{\sigma}_x}$ are taken in the following way:

$$\langle\cdot\rangle_{\boldsymbol{\sigma}_x} = \frac{1}{Z}\sum_{\vec{\boldsymbol{\sigma}}/\boldsymbol{\sigma}_x}(\cdot)e^{-\beta H} . \tag{30}$$

Note that this is not the conditional mean $\langle\cdot|\boldsymbol{\sigma}_x\rangle$, since in that case the normalizing partition sum must also be projected into the state $\boldsymbol{\sigma}_x$.

Since the conditional probability at mean field is normalized ($\sum_{\boldsymbol{\sigma}_x} P(\boldsymbol{\sigma}_x|\vec{\boldsymbol{\mu}}) = 1$), it is important that the correction terms sum up to zero across all states at a lattice site to conserve the normalization. It is easy to see that $\sum_{\boldsymbol{\sigma}_x}\langle\cdot\rangle_{\boldsymbol{\sigma}_x} = \langle\cdot\rangle$. Therefore,

$$\sum_{\boldsymbol{\sigma}_x}\left[\langle\sigma_y^\alpha\sigma_y^\beta\rangle_{\boldsymbol{\sigma}_x} - \langle\sigma_y^\alpha\rangle_{\boldsymbol{\sigma}_x}\langle\sigma_y^\beta\rangle - \langle\sigma_y^\beta\rangle_{\boldsymbol{\sigma}_x}\langle\sigma_y^\alpha\rangle + P(\boldsymbol{\sigma}_x|\vec{\boldsymbol{\mu}})\left(2\langle\sigma_y^\alpha\rangle\langle\sigma_y^\beta\rangle - \langle\sigma_y^\alpha\sigma_y^\beta\rangle\right)\right] \tag{31}$$

$$= \langle\sigma_y^\alpha\sigma_y^\beta\rangle - \langle\sigma_y^\alpha\rangle\langle\sigma_y^\beta\rangle - \langle\sigma_y^\beta\rangle\langle\sigma_y^\alpha\rangle + \left(2\langle\sigma_y^\alpha\rangle\langle\sigma_y^\beta\rangle - \langle\sigma_y^\alpha\sigma_y^\beta\rangle\right) = 0\,. \tag{32}$$

Thus, normalization is conserved by the correction term. Note that this does not ensure that probabilities become negative or greater than one if intrinsic and extrinsic fluctuations are chosen inappropriately.

Calculation of positional information by transfer matrices

Transfer matrices can be used to efficiently compute the partition sum Z of an Ising spin lattice with nearest neighbor interactions. With some modifications this method can be extended to compute all terms in Eq. 29, which can then be used to compute positional information with Eq. 20. The approach described here can be implemented in any programming framework capable of basic matrix algebra.

The entries of a transfer matrix T_x at position x are Boltzmann factors, $\exp(-\beta H(\boldsymbol{\sigma}_x, \boldsymbol{\sigma}_{x+1}))$, where $H(\boldsymbol{\sigma}_x, \boldsymbol{\sigma}_{x+1}) = -\sum_{\alpha=1}^K h_x^\alpha\sigma_x^\alpha - \sum_{\alpha=1}^K\sum_{\beta=\alpha+1}^K J_{\alpha\beta}\sigma_x^\alpha\sigma_x^\beta + \sum_{\alpha=1}^K J_\alpha\sigma_x^\alpha\sigma_{x+1}^\alpha$ is the term of the full Hamiltonian assigned to position x. The entries are ordered such that each row represents a different realization of $\boldsymbol{\sigma}_x$ (such as $\boldsymbol{\sigma}_x = (+1,-1,-1,+1)$) and each column represents a different realization of $\boldsymbol{\sigma}_{x+1}$. For K spins per lattice site the corresponding transfer matrices are of size $2^K\times 2^K$. For example, the transfer matrices for $K=1$ are of the form

$$T_x = \begin{pmatrix} e^{-\beta(-h_x-J)} & e^{-\beta(-h_x+J)} \\ e^{-\beta(h_x+J)} & e^{-\beta(h_x-J)} \end{pmatrix}. \tag{33}$$

For brevity we introduce the notation $T_x = [\{\exp(-\beta H(\boldsymbol{\sigma}_x, \boldsymbol{\sigma}_{x+1}))\}]$. The last lattice site at $x = N$ has no right neighbor and therefore the transfer matrix has the form

$$T_N = \begin{pmatrix} e^{-\beta(-h_x)} \\ e^{-\beta(h_x)} \end{pmatrix}. \tag{34}$$

Then, the partition sum is computed by

$$Z = R_1\cdot\prod_{x=1}^N T_x\,, \tag{35}$$

where $R = 1$ is a row vector of length 2^K with entries of 1.

For the computation of $P(\boldsymbol{\sigma}_x|\vec{\boldsymbol{\mu}})$ for a fixed (mean) input field $\vec{\boldsymbol{\mu}}$ we must sum $P(\vec{\boldsymbol{\sigma}}|\vec{\boldsymbol{\mu}}) = e^{-\beta H}/Z$ over all combination of spin realizations except at lattice site x. This can be written in the form $P(\boldsymbol{\sigma}_x|\vec{\boldsymbol{\mu}}) = Z_{\boldsymbol{\sigma}_x}/Z$, where

$$Z_{\boldsymbol{\sigma}_x} = R_1\cdot\left(\prod_{y=1}^{x-1} T_y\right)\cdot(P_{\boldsymbol{\sigma}}\cdot T_x)\cdot\left(\prod_{y=x+1}^N T_y\right), \tag{36}$$

and the projection matrix

$$P_{\boldsymbol{\sigma}} = \begin{pmatrix} 0 & . & . & . & 0 \\ . & . & & & . \\ . & & 1 & & . \\ . & & & . & . \\ 0 & . & . & . & 0 \end{pmatrix}, \tag{37}$$

which has only one 1 on the diagonal in that row which corresponds to the state $\boldsymbol{\sigma}$ of interest at position x. That means that if the enumeration of states begins with $\boldsymbol{\sigma} = (+1,+1,...,+1)$, then the $P_{\boldsymbol{\sigma}}$ to use in Eq. 36 has an entry 1 at position (1,1) and otherwise 0. The projected transfer matrix $P_{\boldsymbol{\sigma}}\cdot T_x$ only retains entries which correspond to the specific state $\boldsymbol{\sigma}$ at position x.

Eq. 29 also contains the expressions $\langle\sigma_x^\alpha\rangle$, $\langle\sigma_x^\alpha\sigma_x^\beta\rangle$, $\langle\sigma_x^\alpha\rangle_{\boldsymbol{\sigma}_z}$ and $\langle\sigma_x^\alpha\sigma_x^\beta\rangle_{\boldsymbol{\sigma}_z}$. We will briefly discuss the computation of these quantities in the following. The expectation values are computed in the following way:

$$\langle\sigma_x^\alpha\rangle = \frac{1}{Z}\left[R_1\cdot\left(\prod_{y=1}^{x-1}T_y\right)\cdot M_{\sigma_x^\alpha}\cdot\left(\prod_{y=x+1}^{N}T_y\right)\right], \tag{38}$$

$$\langle\sigma_x^\alpha\sigma_x^\beta\rangle = \frac{1}{Z}\left[R_1\cdot\left(\prod_{y=1}^{x-1}T_y\right)\cdot M_{\sigma_x^\alpha\sigma_x^\beta}\cdot\left(\prod_{y=x+1}^{N}T_y\right)\right]. \tag{39}$$

In the notation introduced above, the matrices assigned to position x can be written as $M_{\sigma_x^\alpha} = [\{\sigma_x^\alpha\exp(-\beta H(\boldsymbol{\sigma}_x,\boldsymbol{\sigma}_{x+1}))\}]$ and $M_{\sigma_x^\alpha\sigma_x^\beta} = [\{\sigma_x^\alpha\sigma_x^\beta\exp(-\beta H(\boldsymbol{\sigma}_x,\boldsymbol{\sigma}_{x+1}))\}]$. That is, the rows of these matrices have signs depending on which sign σ_x^α or $\sigma_x^\alpha\sigma_x^\beta$ have in the corresponding state.

The computation of the modified expectation values $\langle\sigma_x^\alpha\rangle_{\boldsymbol{\sigma}_z}$ and $\langle\sigma_x^\alpha\sigma_x^\beta\rangle_{\boldsymbol{\sigma}_z}$ follows the same principe, but with position z projected into a specific state:

$$\langle\sigma_x^\alpha\rangle_{\boldsymbol{\sigma}_z} = \frac{1}{Z}\left[R_1\cdot\left(\prod_{y=1}^{x-1}T_y\right)\cdot M_{\sigma_x^\alpha}\cdot\left(\prod_{y=x+1}^{z-1}T_y\right)\cdot(P_{\boldsymbol{\sigma}}\cdot T_z)\left(\prod_{y=z+1}^{N}T_y\right)\right], \tag{40}$$

$$\langle\sigma_x^\alpha\sigma_x^\beta\rangle_{\boldsymbol{\sigma}_z} = \frac{1}{Z}\left[R_1\cdot\left(\prod_{y=1}^{x-1}T_y\right)\cdot M_{\sigma_x^\alpha\sigma_x^\beta}\cdot\left(\prod_{y=x+1}^{z-1}T_y\right)\cdot(P_{\boldsymbol{\sigma}}\cdot T_z)\left(\prod_{y=z+1}^{N}T_y\right)\right]. \tag{41}$$

I. MONTE-CARLO SIMULATION AND STOCHASTIC OPTIMIZATION

In the case of long range interactions between spins, the approach described above is no longer applicable and we have to resort to sampling of Monte-Carlo simulations. For the description of the algorithm we think of the K spin rows with respectively N lattice sites as concatenated to a single row vector $\vec{\sigma}$ of length $N_{\text{tot}} = K\times N$. However, we keep in mind that the complete position dependent spin state $\boldsymbol{\sigma_x}$ is comprised of entries in $\vec{\sigma}$, which are N vector positions apart. The inputs for the simulation are the intrinsic noise level η, a row vector $\vec{h}$ containing N_{tot} fields and a symmetric $N_{\text{tot}}\times N_{\text{tot}}$ matrix J containing the pairwise coupling strengths between spins. The Hamiltonian of a specific spin configuration is

$$H(\vec{\sigma}) = -\vec{h}\cdot\vec{\sigma}^T - \vec{\sigma}\cdot J\cdot\vec{\sigma}^T. \tag{42}$$

For the estimation of $P(\boldsymbol{\sigma}|x)$ we count the occurrences of each state $\boldsymbol{\sigma}$ at each position x during the simulation in a $(2^K)\times N$ matrix $\mathcal{S}$. The simulation proceeds in the standard Monte-Carlo form:

1. Initialize $\vec{\sigma}$ in a random or assigned start configuration; set counter to 0.
2. Compute $H = H(\vec{\sigma})$.
3. Generate new proposal configuration $\vec{\sigma}_{\text{new}}$ by flipping one spin at random.
4. Compute proposal Hamiltonian $H_{\text{new}} = H(\vec{\sigma}_{\text{new}})$.
5. Accept $\vec{\sigma} = \vec{\sigma}_{\text{new}}$ and $H = H_{\text{new}}$ with probability $\min(1, \exp((H - H_{\text{new}})/\eta))$.
6. For each position x, increment the entry in $\mathcal{S}$ corresponding to the current state by 1.
7. Increment counter by 1.
8. Proceed from 3. until termination criterion is fulfilled.
9. For an estimation $P(\boldsymbol{\sigma}|x)$, divide each entry in $\mathcal{S}$ by counter.

If the intrinsic noise level η is low, the energy landscape often contains local minima which are separated by multiple spin flips with low probability. Therefore, if the system is randomly initialized, it likely that it stays in the vicinity of a local minimum and never reaches the state with the lowest energy. To avoid this problem, we perform a "simulated annealing" step prior to sampling: we start from high noise level and decrease η stepwise to the desired noise level, while letting the system equilibrate between each step. For the stochastic optimization of system parameters we also use simulated annealing.
