# Supplementary material for: Beyond the French Flag Model: Exploiting Spatial and Gene Regulatory Interactions for Positional Information
Source: PLoS One. 2016 Sep 27;11(9):e0163628. doi: 10.1371/journal.pone.0163628 (PMC5038966; doi:10.1371/journal.pone.0163628)
Supplement: S3 Appendix — The positional information in a discrete morphogen field with Gaussian noise is computed. (PDF) [file pone.0163628.s003.pdf]

Computation of positional information in a discrete morphogen field

Patrick Hillenbrand, Ulrich Gerland, and Gašper Tkačik

Computation of the positional information $I(m;x)$ in a discrete morphogen field $\vec{m}$ under the assumptions described in the main text is straightforward. Since we assume the Gaussian fluctuation in the morphogen field to be positionally independent, $P(m|x)$ is a Gaussian distribution $\mathcal{G}$ with mean value $\mu(x)$ and standard deviation ν. Further assuming a flat prior distribution, $P(x) = 1/N$, the positional information is

$$I(m;x) = \frac{1}{N}\sum_{x=1}^{N}\int_{-\infty}^{\infty} dm\,\mathcal{G}(m;\mu(x),\nu)\log\left[\frac{\mathcal{G}(m;\mu(x),\nu)}{\frac{1}{N}\sum_{x=1}^{N}\mathcal{G}(m;\mu(x),\nu)}\right]. \quad (1)$$

This expression can be numerically evaluated for arbitrary mean morphogen profiles $\mu(x)$.
